# Pilot Study on Nucleation-Induced Pelleting Coagulation in Treatment of High-Algae Surface Water: Coagulant Dosage and Hydraulic Loading Optimization

**DOI:** 10.3390/toxics13060418

**Published:** 2025-05-22

**Authors:** Xiangxuan Xing, Tinglin Huang, Ruizhu Hu, Kai Li

**Affiliations:** 1 School of Environmental and Municipal Engineering, Xi’an University of Architecture and Technology, Xi’an 710055, China; xtt851228@126.com (X.X.); huruizhu@xauat.edu.cn (R.H.); likai@xauat.edu.cn (K.L.); 2 Shaanxi Provincial Collaborative Innovation Center for Water Pollution Control and Water Quality Safety, Xi’an University of Architecture and Technology, Xi’an 710055, China

**Keywords:** circulating pelletized fluidized bed, high-algae water, organic matter removal, disinfection byproducts, water quality safety

## Abstract

This study proposes a circulating pelletized fluidized bed (CPFB) with micro-sand loading for treating high-algae surface water. Key operational parameters (coagulant dosage, flow rate) were optimized to simultaneously remove algae, turbidity, and disinfection byproduct precursors. Results revealed that 20 mg/L polyaluminum chloride (PACl) and 0.8 mg/L PAM achieved optimal removal of algae (density removal > 80%) and organic matter. The fluidized bed exhibited robust performance across algal species, with the highest dichloroacetonitrile (DCAN) precursor removal of 66.20%, demonstrating superior efficiency for trihalomethane precursors over haloacetic acids. These findings provide critical operational guidance for high-algae water treatment using fluidized beds.

## 1. Introduction

With the intensification of human activities, the issue of eutrophication in lakes and reservoirs has become increasingly severe, leading to more frequent cyanobacterial blooms [1]. In recent years, several significant water bodies in China, including Taihu Lake [2], Chaohu Lake, and Dianchi Lake [3], have faced large-scale cyanobacterial blooms, which have triggered urban water supply crises. The presence of algal cells and their metabolites in water has posed numerous challenges to drinking water treatment and water quality safety. This includes an increase in precursors of disinfection byproducts (DBPs), significantly elevating the potential for DBP formation in treated water [4] and raising concerns about public health [5].

Conventional drinking water treatment methods, such as coagulation–sedimentation–filtration, are effective in removing some high-molecular-weight organic matter and colloidal particles [6]. However, they face difficulties in removing organic matter with molecular weights below 10 kDa—such as aldehydes (e.g., formaldehyde), carboxylic acids (e.g., acetic acid), and aromatic compounds (e.g., phenols)—proves difficult. These low-molecular-weight organic compounds are major contributors to DBP formation, particularly trihalomethanes (THMs) and haloacetic acids (HAAs), both of which pose serious health risks [7].

The circulating pelletized fluidized bed (CPFB) has emerged as a highly efficient solid–liquid separation system that integrates coagulation, clarification, sedimentation, and particle circulation [8]. Based on the theory of pellet flocculation, it has undergone several structural improvements [9]. This system offers significant advantages, including high treatment capacity, short hydraulic retention time, compact size, and strong adaptability to variable water quality [10]. CPFB technology has been widely implemented in various water treatment plants across China [11].

Previous studies on pellet flocculation technology applied to algae-rich water include the work by Huang Tinglin and Tai Chuanmin et al., who conducted a pilot study on high-algae water from the Tangyu Reservoir in Xi’an, China, during the summer. They successfully demonstrated the feasibility of a micro-sand-enhanced pellet flocculation process, achieving algae removal rates of up to 80%, COD reduction of 80%, and turbidity control below 1.5 NTU [12]. The Actiflo clarifier, developed by the French water company Veolia, employs similar methods, using high-molecular-weight flocculants and micro-sand to enhance colloid separation from low-concentration raw water [13]. Furthermore, research by Jin Xin et al. on nucleation-induced pelleting coagulation (NPC) has highlighted the effectiveness of this process in treating fracturing wastewater by removing dissolved organic matter [14]. The removal of both algae and organic matter depends heavily on the optimal dosage of coagulants [15]. Studies have shown that combining coagulants with natural mineral additives, such as diatomite, can significantly enhance algae removal efficiency, with optimized dosages achieving over 90% removal rates [16]. Emerging techniques, such as ultrasound-assisted coagulation [17], have been shown to improve cell disruption and floc formation, further enhancing removal performance. Electrocoagulation, another promising alternative, has demonstrated effective removal of algal organic matter (AOM) while producing less sludge, making it suitable for decentralized treatment systems [18].

Despite these promising technologies, challenges persist in optimizing coagulant dosages and minimizing the environmental impact of residual chemicals. For instance, excessive PACl residue can lead to aluminum accumulation in aquatic ecosystems, posing risks to aquatic life [19]. Similarly, excessive PAM can harm microorganisms and other aquatic organisms. Hence, careful monitoring residual concentrations and optimizing chemical dosing is essential for ensuring both efficient treatment and environmental safety [20].

This study aims to further optimize the coagulation processes for treating algae-laden water using the CPFB system. The primary goal is to assess the removal efficiency of turbidity, algae, and algae-derived organic matter under various operational conditions and to identify the optimal coagulant dosages that balance treatment efficiency with operational costs.

## 2. Materials and Methods

### 2.1. Raw Water

The raw water was collected from a water treatment plant in western China. Key characteristics of the water quality during the experimental period are summarized in Table 1. Notably, turbidity exhibited extreme variability (2–5000 NTU), spanning both low-turbidity [21] and high-turbidity [22] ranges. To address this variability, this experiment primarily targeted effluent turbidity, CODMn, and algal removal rate as critical performance indicators under varying influent turbidity conditions. These metrics were used to determine optimal process control parameters, specifically coagulant dosage, coagulant-aid dosage, and impeller rotation rate, which provided actionable guidance for subsequent full-scale applications.

### 2.2. Experimental Device

**Figure 1:**
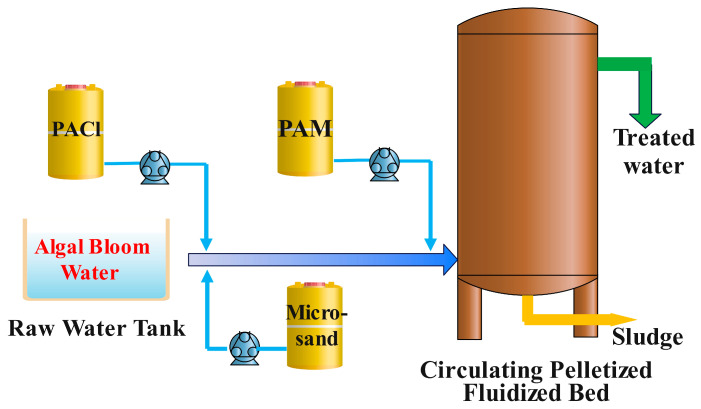
Schematic of circulating granulation fluidized bed.

The pilot test setup is shown in Figure 1. It mainly consists of a water distribution system, a mixing device, a tubular reactor, a slurry tank, a sand feeding pump, and a pelletized fluidized bed. The micro-sand-enhanced pelletized flocculation process integrates reaction, sedimentation, and concentration into one compact structure, simplifying operational complexity. The pilot equipment used in the experiment has a diameter of 1.3 m and a height of 4.5 m. The reaction tube is 20 m long with a diameter of 25 mm. The clear water zone is located at the top of the equipment, with a height of 1.5 m. After adding the coagulant to the raw water, it is vigorously mixed in a static mixer to destabilize the colloidal particles in the water. Following the static mixer, micro-sand is added as nuclei for the destabilized colloids (quartz sand needs to be added in advance when treating low-turbidity water to quickly form a suspended layer), forming ideal initial particles in the tubular reactor. Before entering the pelletized fluidized bed, polyacrylamide is added. The initial particles further coagulate and grow through the adsorption and bridging effects of the polymer and the various densification forces in the pelletized flocculation zone of the fluidized bed, forming high-density pelletized flocs (pellets). The pellets achieve solid–liquid separation in the clarification zone, with clear water discharged from the top and sludge entering the outer cylinder sludge concentration zone. Micro-sand is recycled for reuse.

### 2.3. Preparation of Chemicals and Micro-Sand

The coagulant used was polyaluminum chloride (PACl) produced by Tongxin Chemical Co., Ltd. in Xianyang, Shaanxi, China, with a preparation concentration of (30%). The coagulant aid was AN932 food-grade anionic polyacrylamide (PAM, solid content ≥ 90%, acrylamide monomer content ≤ 0.025%) produced by SNF France. The micro-sand used had a particle size of 80–120 mesh (≈125–180 μm), with a preparation concentration of 5–20 g/L.

### 2.4. Analytical Methods

Turbidity was measured using a turbidimeter (2100N, Hach, Loveland, CO, USA). UV_254_ was measured using a UV–visible spectrophotometer (UV-1600, Hach). COD_Mn_ was determined by the acidic potassium permanganate titration method. DOC was measured using a total organic carbon analyzer (Shimadzu 40L, Tokyo, Japan). The determination of disinfection byproduct formation potential followed the USEPA Method 551, using gas chromatography [23].

## 3. Results and Discussion

### 3.1. Determination of the Optimal Dosage of Coagulant

#### 3.1.1. Determination of PACl Dosage

**Figure 2:**
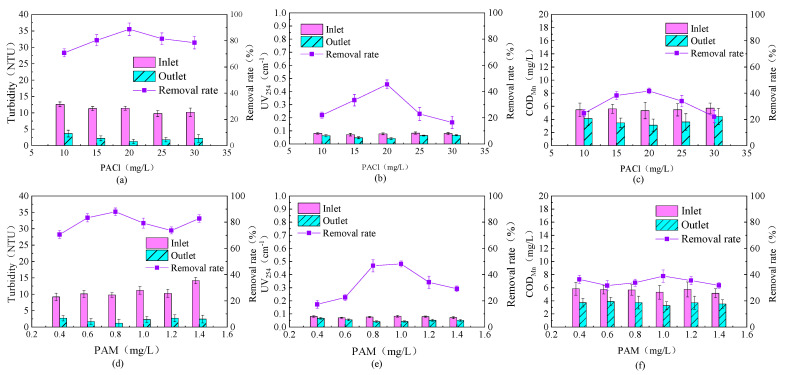
Effects of different PACl (**a**–**c**) and PAM (**d**–**f**) dosages on the removal of organic matter in source water. (Rising flow rate: 55 m/h; stirring speed: 6 r/min; (**a**–**c**) PAM dosage 0.6 mg/L; (**d**–**f**) PACl dosage 20 mg/L).

The experimental results of the effect of PACl dosage on the removal of organic matter from source water by the fluidized bed are shown in Figure 2a–c. As can be seen from Figure 2a–c, as the PACl dosage increases, the removal rate of effluent turbidity first rises and then falls. Similarly, the removal rates of UV_254_ and COD_Mn_ in the effluent also show the same trend with increasing PACl dosage. This indicates that the content of organic matter in the effluent from the fluidized bed is positively correlated with the effluent turbidity.

Under the operating conditions, when the PACl dosage was 20 mg/L, the fluidized bed achieved the best removal efficiency for turbidity, UV_254_, and COD_Mn_, with removal rates of 88.77%, 45.45%, and 41.68%, respectively. Compared to traditional coagulation experiments, the fluidized bed required lower chemical dosages to achieve the same level of turbidity and organic matter removal, which may be related to the formation of aggregates in the fluidized bed.

**Figure 3:**
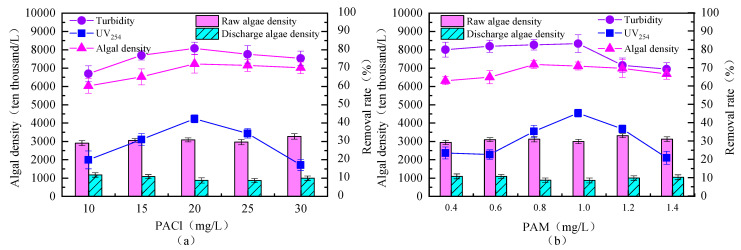
Effects of different PACl (**a**) and PAM (**b**) dosages on the removal of algae in source water. (Rising flow rate: 55 m/h; stirring speed: 6 r/min; (**a**) PAM dosage 0.6 mg/L; (**b**) PACl dosage 20 mg/L).

The effect of PACl dosage on the removal of algae from source water by the fluidized bed is shown in Figure 3a. It can be observed that as the PACl dosage increased, the algal density in the effluent first decreased and then slowly increased. When the PACl dosage was 20 mg/L, the highest algal density removal rate of 71.63% was achieved. At this dosage, the removal rates for turbidity and UV_254_ were also the highest, at 80.12% and 41.67%, respectively.

The removal rate of algal density showed an initial increase followed by a decrease, which may be due to excessive PACl dosage altering the surface charge of the aggregates, or the increased PACl dosage affecting the effective growth of the floc, thereby reducing the algae removal efficiency [24].

The observed improvement in removal efficiency with increasing coagulant dosage is consistent with the classical mechanisms of charge neutralization and sweep flocculation [25]. Higher dosages promote the formation of precipitates, which facilitate the enmeshment and aggregation of algal cells and organic matter. However, when the dosage exceeds the optimal threshold, particle restabilization or excessive sludge generation may occur, as noted by Wang [24]. The optimal coagulant dosage identified in this study achieves a favorable balance between treatment performance and operational cost, aligning well with previous research on aluminum-based coagulants for algae-laden waters [26]. Furthermore, the enhanced particle–floc collision frequency within the CPFB system allows for effective removal even at relatively lower dosages, underscoring the system’s efficiency compared to conventional coagulation processes. Based on the removal effects of both organic matter and algae, the optimal PACl dosage was determined to be 20 mg/L.

#### 3.1.2. Determination of PAM Dosage

The experimental results of the effect of PAM dosage on the removal of organic matter in source water by the fluidized bed are shown in Figure 2d–f. It can be observed that as the PAM dosage increased from 0.4 mg/L to 0.8 mg/L, the turbidity of the effluent showed a decreasing trend, reaching a minimum of 1.18 NTU, and the removal rate also showed an increasing trend. However, when the PAM dosage continued to increase, the effluent turbidity significantly rose, and the removal rate exhibited an unstable curve. As the PAM dosage increased, the UV_254_ content in the effluent first decreased and then increased, with the removal rate also showing an initial increase followed by a gradual decrease. With the increase in PAM dosage, the COD_Mn_ content in the effluent remained relatively stable, and the removal rate showed a relatively stable wave-like pattern. The results indicate that changes in PAM dosage have a certain impact on the effluent turbidity but do not significantly affect the removal of organic matter. The bridging effect of PAM causes the flocs in the fluidized bed to grow larger, but excessive PAM dosage may reduce the density of the aggregates, which adversely affects adsorption and sedimentation.

Based on the optimal PACl dosage of 20 mg/L, the effect of PAM dosage on the removal of algae from source water by the fluidized bed was studied, and the experimental results are shown in Figure 3b. It can be observed that, in source water with high algal content, as the PAM dosage increased, the algal density in the effluent first decreased and then slowly increased, with the removal rate showing a corresponding trend. When the PAM dosage was 0.8 mg/L, the algal density removal rate reached 71.74%, and the removal rates for turbidity and UV_254_ were 82.45% and 35.22%, respectively. These results are similar to those of the PACl experiments, possibly because the increased PAM dosage caused poor internal circulation in the fluidized bed, affecting the growth of the flocs and thereby reducing the algae removal efficiency.

The addition of a polymer flocculant significantly improved pellet formation, indicating that adsorption bridging played a vital role in enhancing floc size and cohesion. These findings corroborate earlier studies that demonstrated the synergistic effects of coagulant and flocculant combinations in enhancing sludge settleability and floc strength [27]. The formation of denser, more uniform pellets within the fluidized bed also suggests improved shear resistance and mechanical stability, crucial for sustained operation. Moreover, the observed impact on pellet size distribution indicates that flocculant dose must be carefully tuned to avoid overdosing, which could lead to overly large, fragile aggregates prone to breakage—a challenge previously reported in similar systems [28].

Considering the removal effects of both organic matter and algae, as well as treatment costs, the optimal PAM dosage was determined to be 0.8 mg/L when the PACl dosage was 20 mg/L.

### 3.2. Determination of Optimal Upflow Velocity

**Figure 4:**
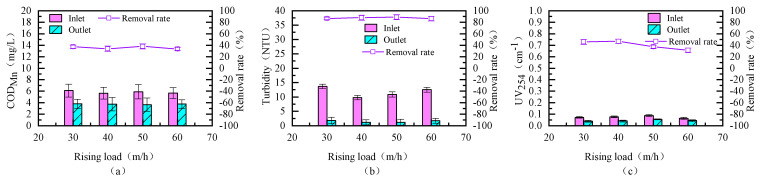
Removal efficiency of organic matter in source water under different upflow loadings ((**a**–**c**) COD_Mn_, Turbidity, UV_254_; PACl: 20 mg/L; PAM: 0.8 mg/L).

We further investigated the impact of upflow loading on the removal of organic matter from source water by the fluidized bed. The experimental results are shown in Figure 4. As can be seen from the figure, the effect of upflow loading on the effluent turbidity and organic matter removal is relatively weak. When the upflow loading is between 30 m/h and 60 m/h, the removal rates for turbidity, UV_254_, and COD_Mn_ are 86.36–88.77%, 31.25–46.7%, and 33.8–37.9%, respectively. The results indicate that the fluidized bed has a certain level of impact resistance and stability in response to varying water flow rates.

**Figure 5:**
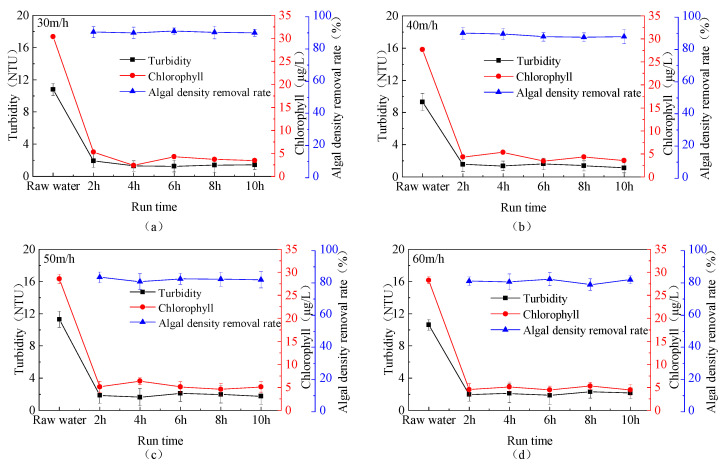
Removal efficiency of total algae cell count in source water by the fluidized bed under different upflow loadings (PAC: 20 mg/L; PAM: 0.8 mg/L; stirring speed: 6 r/min; (**a**) upflow loadings: 30 m/h, (**b**) upflow loadings: 40 m/h, (**c**) upflow loadings: 50 m/h, (**d**) upflow loadings: 60 m/h).

The removal efficiency of algae density and chlorophyll in source water by the fluidized bed under different upflow loadings is shown in Figure 5. As can be seen from the figure, as the upflow loading increases, the removal efficiency of algae density gradually decreases, and the chlorophyll content in the effluent also increases. When the upflow loading is 30 m/h, the algae density removal rate is 89.94–90.98%; at 40 m/h, it is 87.6–90%; at 50 m/h, it is 80.78–83.48%; and, at 60 m/h, it is 78.93–82.35%. This trend may be attributed to the fact that increased upflow loading causes algal cells to break under the shear force of the water flow and collisions with various particles, making it challenging for the fluidized bed to effectively remove them. Therefore, for optimal treatment of algae-rich source water, the fluidized bed should be operated at a low upflow loading while ensuring normal and stable operation.

**Figure 6:**
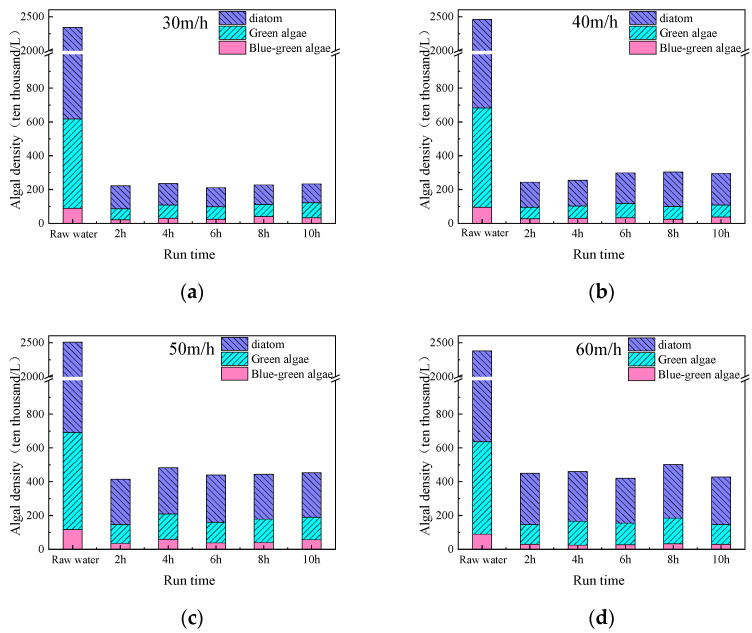
Removal efficiency of different types of algae in source water by the fluidized bed under different upflow loadings (PAC: 20 mg/L; PAM: 0.8 mg/L; stirring speed: 6 r/min; (**a**) upflow loadings: 30 m/h, (**b**) upflow loadings: 40 m/h, (**c**) upflow loadings: 50 m/h, (**d**) upflow loadings: 60 m/h).

Further studies were conducted on the removal efficiency of different types of algae in source water using a fluidized bed under varying upflow loadings. The results, as shown in Figure 6, indicate that the source water contains a high proportion of diatoms, and the fluidized bed has a significant removal effect on diatoms, green algae, and cyanobacteria. This highlights the effectiveness of the fluidized bed in removing algae from source water. It can be observed that as the upflow loading of the fluidized bed increases, the proportion of diatoms in the effluent gradually increases, indicating a decrease in the removal efficiency of diatoms. A similar trend is observed for green algae, while cyanobacteria do not show a clear trend. This discrepancy may be attributed to the higher proportion of diatoms and green algae in the source water, which makes the effluent quality more sensitive to upflow loading variations.

Upflow velocity plays a crucial role in the hydrodynamic environment of the fluidized bed, balancing fluidization and particle retention. Our findings confirm that moderate velocities enhance pellet circulation and contact without inducing washout, which is in line with the results of pilot studies by Huang [29]. Excessively low velocities can cause sedimentation and clogging, while Excessively high velocities may reduce residence time and promote pellet disintegration. The optimal velocity range identified here supports both efficient removal and stable pellet morphology [30]. These results underscore the critical importance of hydraulic optimization in maintaining long-term reactor performance under variable water quality conditions. Proper optimization ensures that the fluidized bed operates effectively, maintaining high removal efficiencies and stable operation, even as environmental conditions change.

### 3.3. Water Treatment Efficiency Under Stable NPC Operation

#### 3.3.1. Removal Efficiency of Algae and Organic Matter During Continuous Fluidized Bed Operation

**Figure 7:**
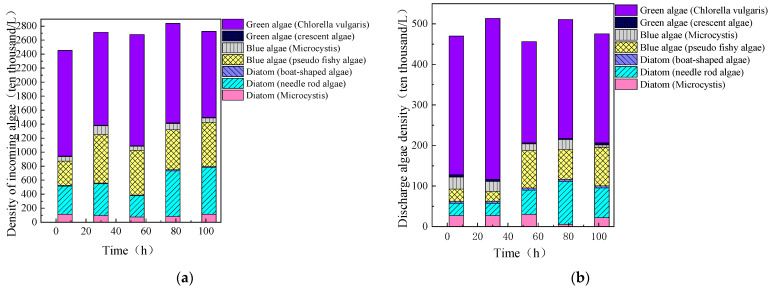
Density of various algae in the influent (**a**) and effluent (**b**) during continuous fluidized bed operation (PAC: 20 mg/L; PAM: 0.8 mg/L; stirring speed: 6 rpm).

The stable operation of the fluidized bed is a crucial parameter for its ability to continuously and effectively remove algae. Ammonia nitrogen and total phosphorus (TP) are among the key components leading to water eutrophication, which can cause the proliferation of cyanobacteria and green algae. Therefore, this study investigated the removal efficiency of algae and organic matter in water during 100 h of continuous fluidized bed operation. As shown in Figure 7, Chlorella (a type of green algae) was the dominant species in the source water, accounting for more than 50% of the total algal density, followed by Pseudanabaena (a type of cyanobacteria) and Synedra (a type of diatom). The fluidized bed demonstrated a consistent and effective removal of algae from the source water. The proportion of Chlorella in the effluent was higher than in the influent, but, as the fluidized bed continued to operate, the proportion of Chlorella in the effluent gradually decreased, although it remained the dominant species. This may be because the removal rate of Chlorella by the fluidized bed was not as high as that of other algae, and, over time, the removal rates of various algae tended to converge.

**Figure 8:**
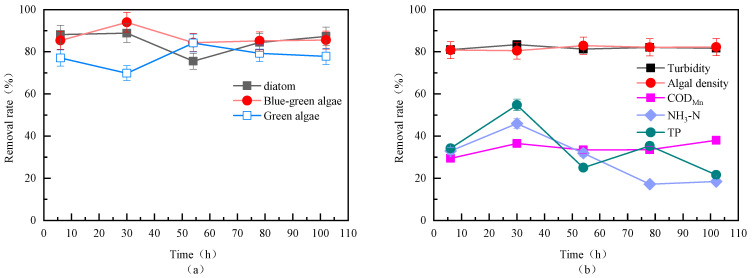
Removal efficiency of different algae (**a**) and organic matter (**b**) in the source water during continuous fluidized bed operation (PAC: 20 mg/L; PAM: 0.8 mg/L; stirring speed: 6 rpm).

Based on the above observations, this study further evaluated the removal efficiency of different algae species in the source water during continuous fluidized bed operation, as shown in Figure 8a. The results indicate that the fluidized bed had a good removal effect on algae. Over time, the removal rates of various algae gradually converged, likely due to the stable and uniform pore structure of the floc particles formed during the continuous operation of the fluidized bed, leading to more consistent removal rates for different algae. The removal rates for diatoms, cyanobacteria, and green algae were 87.33%, 85.55%, and 77.8%, respectively.

As shown in Figure 8b, the removal rates of turbidity and algal density remained relatively stable over time, with comparable removal rates ranging from 80.48% to 83.40%. Combined with Figure 8a, it can be concluded that the overall removal rate of algae by the fluidized bed was consistent, while the removal rates for diatoms, green algae, and cyanobacteria gradually converged over time. The removal rate of COD_Mn_ was also relatively stable, ranging from 29.38% to 38.04%. However, the removal rates of ammonia nitrogen and TP fluctuated significantly, showing an initial increase followed by a gradual decrease, eventually stabilizing at 18.46% and 21.62%, respectively. This may be due to the gradual release of ammonia nitrogen and TP from the floc particles over time, leading to a decrease in their removal rates in the effluent [31]. The removal effect of ammonia nitrogen was similar to the traditional coagulation methods.

#### 3.3.2. Removal Efficiency of Different Algae During Continuous Fluidized Bed Operation

(1) Removal Efficiency of Diatoms

**Figure 9:**
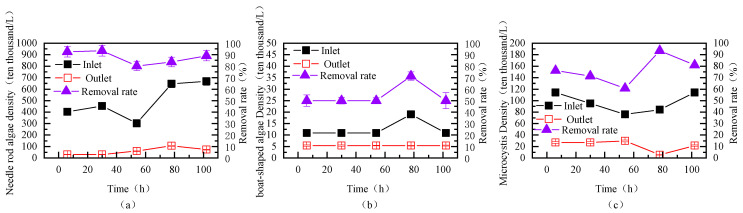
Removal efficiency of different diatoms in the source water during continuous fluidized bed operation (PAC: 20 mg/L; PAM: 0.8 mg/L; stirring speed: 6 rpm; (**a**) Needle rod algae, (**b**) Boat-shaped algae, (**c**) Microcystis).

This study investigated the removal efficiency of different diatoms in the source water by the fluidized bed, as shown in Figure 9. The main diatoms detected in the source water were Cyclotella, Synedra, and Navicula, with Synedra having the highest density, reaching up to 6,683,820 cells/L. The fluidized bed showed a relatively stable removal rate for Synedra, with a maximum of 93.41%. The removal rate for Cyclotella gradually increased over time, ultimately reaching a maximum of 93.53%, while the removal rate for Navicula ranged between 50% and 71.43%.

(2) Removal Efficiency of Cyanobacteria

**Figure 10:**
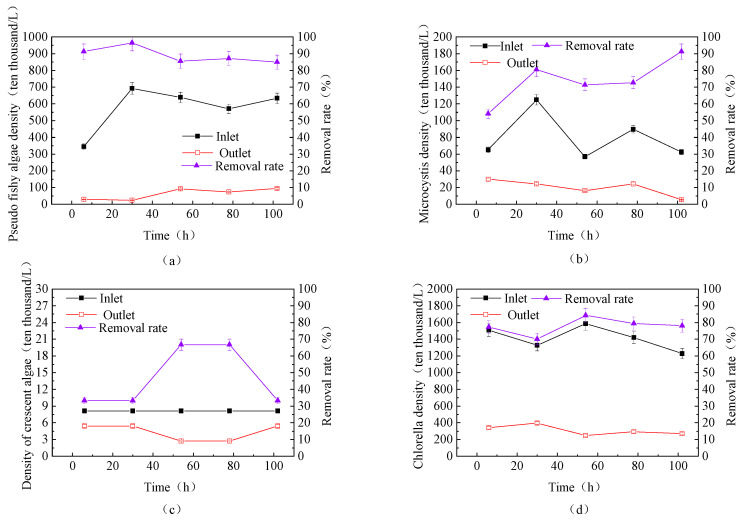
Removal efficiency of different cyanobacteria (**a,b**) and green algae (**c**,**d**) in the source water during continuous fluidized bed operation (PAC: 20 mg/L; PAM: 0.8 mg/L; stirring speed: 6 rpm).

This study also examined the removal efficiency of different cyanobacteria in the source water using the fluidized bed, as shown in Figure 10a. The main cyanobacteria in the source water were Pseudanabaena and Microcystis, with Pseudanabaena reaching a maximum density of 6,928,350 cells/L and Microcystis reaching a maximum density of 1,249,820 cells/L. The removal rate of Pseudanabaena by the fluidized bed was relatively stable and high, ranging from 84.98% to 96.47%. As the fluidized bed continued to operate, the removal rate of Microcystis gradually increased, reaching 91.3%, and the density of Microcystis in the effluent continued to decrease.

(3) Removal Efficiency of Green Algae

This study further investigated the removal efficiency of different green algae in the source water using the fluidized bed, as shown in Figure 10b. The main green algae in the source water were Chlorella, with a maximum density of 15,867,280 cells/L. The removal rate of Chlorella by the fluidized bed ranged from 69.86% to 84.16%, while the removal rate of Selenastrum ranged from 33.33% to 66.66%.

Comparing the removal rates of the three dominant algae—Synedra, Pseudanabaena, and Chlorella—it can be observed that the removal rate of Chlorella was the lowest, which is consistent with the speculation in Section 3.3.1. Although the fluidized bed’s removal rate for Chlorella was not as high as those of other dominant algae, but, over time, the removal rates for all various algae gradually converged over time.

The CPFB system maintained high removal efficiencies during extended operation, demonstrating excellent process stability and resilience against fluctuations in influent quality. This is particularly important for algae-rich surface waters, where sudden algal blooms can challenge conventional systems [32]. Compared to batch or sedimentation-based treatments, the fluidized bed provides continuous mixing and pellet self-renewal, both of which reduce maintenance needs and enhance robustness [33]. The long-term results presented in this study represent one of the few to document sustained performance over several weeks, providing valuable operational data to support the technology’s real-world applicability.

### 3.4. Water Quality Safety of NPC-Treated Algae-Rich Water

#### 3.4.1. Changes in Disinfection Byproduct Formation Potential Before and After Fluidized Bed Treatment

**Figure 11:**
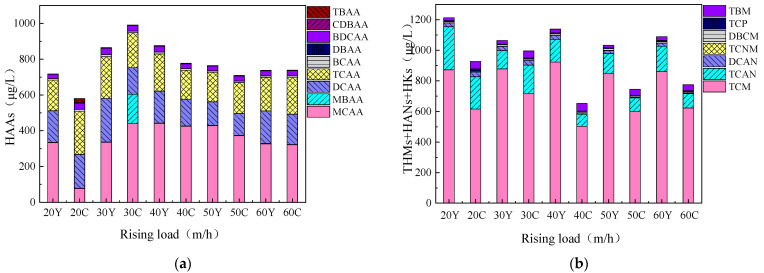
Formation potential of disinfection byproducts before and after fluidized bed treatment under different upflow velocities. Y: raw water; C: effluent; PAC: 20 mg/L; PAM: 0.8 mg/L; stirring speed: 6 r/min; (**a**) HAAs, (**b**) THMs+HANs+HKs.

THMs (trihalomethanes) and HAAs (haloacetic acids) are among the DBPs (disinfection byproducts) that have been included in the control standards for drinking water quality in most countries, making them essential regulatory indicators [34]. This study investigated the changes in the formation potential of four types of carbon-containing disinfection byproducts—THMs, HAAs, HANs (haloacetonitriles), and HKs (haloketones)—before and after fluidized bed treatment under different upflow velocities. The results are shown in Figure 11, reveal that for HAAs, the formation potential of chlorinated byproducts such as MCAA, DCAA, and TCAA is the highest. Among THMs, TCM and TCAN in HANs exhibit the highest formation potential. The formation potential of HKs is the lowest, followed by HANs, while THMs show the highest formation potential overall. Although the formation of HAAs is lower than that of THMs, HAAs are more toxic. The fluidized bed shows better removal efficiency for the formation potential of THMs compared to HAAs. At an upflow velocity of 40 m/h, the fluidized bed achieves the best removal of THMs formation potential. The results indicate that the fluidized bed can effectively remove the precursors of THMs, which is contrary to the performance of conventional processes. Conventional processes are more effective in removing the precursors of HAAs than THMs. This is because high-molecular-weight organic matter serves as the precursor for HAAs, and coagulation is highly effective in removing high-molecular-weight organic matter but less effective for low-molecular-weight organic matter [35].

#### 3.4.2. Removal Efficiency of Fluidized Bed for Different Types of Disinfection Byproduct Formation Potential

**Figure 12:**
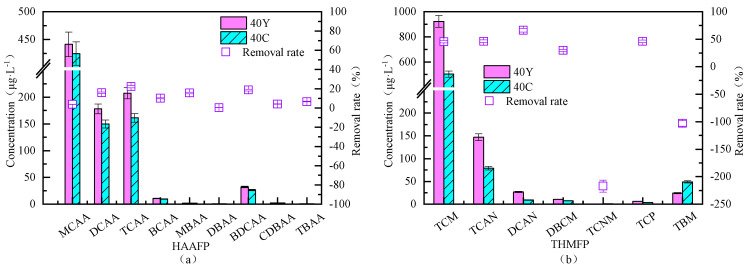
Formation potential of different types of disinfection byproducts before and after fluidized bed treatment. Y: raw water; C: effluent; PAC: 20 mg/L; PAM: 0.8 mg/L; stirring speed: 6 r/min; (**a**) HAAs, (**b**) THMs+HANs+HKs.

The influent and effluent of the fluidized bed operating at an upflow velocity of 40 m/h were analyzed for analysis of disinfection byproduct formation potential. The results are shown in Figure 12, indicate that the fluidized bed achieved a removal rate of only 0.399–22.15% for haloacetic acid (HAA) formation potential, whereas the removal rate of THMs formation potential ranged from 29.74% to 45.52%. Notably, The removal rates of TCNM and TBM formation potential were negative, which may result from an increase in precursors for these byproducts or transformations of other precursors, leading to a decrease in other byproducts’ formation potential and a concurrent increase in TCNM and TBM formation potential. For HANs, the fluidized bed achieved the highest removal rate (66.20%) for dichloroacetonitrile (DCAN), reducing its formation potential in the effluent to 9.05 μg/L. In the case of haloketones (HKs), the removal rates for dibromochloromethane (DBCM) and trichloropropanone (TCP) were 29.75% and 46.09%, respectively.

Ensuring water quality safety is essential when applying the NPC (Nucleation Pelletized Coagulation) system to algae-rich surface waters. The results of this study indicate that NPC-treated effluent consistently meets drinking water standards regarding turbidity, algal cell count, and organic matter concentration. This highlights the system’s capability to effectively remove algal cells and reduce organic precursors that could otherwise lead to disinfection byproduct (DBP) formation, particularly during downstream chlorination [36,37].

The superior performance of the NPC system can be attributed to its ability to generate compact, stable pellets that efficiently trap algal cells and associated organic residues. Compared to conventional coagulation–sedimentation methods, which often struggle with residual algal content, the continuous pellet renewal process minimizes the risk of toxin release from disrupted cells [38]. This feature is particularly advantageous in managing seasonal algal blooms, where high biomass loads might compromise water quality.

Furthermore, the consistent reduction in turbidity and organic matter not only improves the visual quality of treated water but also decreases the formation potential of hazardous DBPs, such as trihalomethanes (THMs) and haloacetic acids (HAAs) [39]. These results support the NPC system’s potential as a robust and reliable solution for treating algae-contaminated waters, especially in areas reliant on chlorination for disinfection.

## 4. Conclusions

This study investigated the application of a CPFB system, enhanced with micro-sand loading, for the treatment of algae-rich surface water. The findings demonstrate that the system exhibits strong performance in removing turbidity, algae, and organic matter. By optimizing the dosages of coagulants and coagulant aids and precisely controlling the upflow velocity, a highly efficient and stable treatment process was achieved. Long-term operation confirmed the system’s ability to consistently and effectively remove various algal species, thereby ensuring the reliability and safety of the treated water. This research not only confirms the technical feasibility of CPFB technology for addressing high-algae water conditions but also offers a valuable theoretical foundation and technical guidance for its practical implementation. Future research will focus on scaling up the system and exploring its integration with other treatment technologies to improve overall performance and economic viability.

## Figures and Tables

**Table 1:** Water quality characteristics of experimental raw water.

pH	Turbidity/NTU	COD_Mn_/mg·L^−1^	Algal Density (×10^4^ cells/L)	Temperature (°C)	Zeta Potential (mV)
7.73~8.45	2~5000	4.9~6.6	2680~3290	9–17	−22.8~−23.8

## Data Availability

The data presented in this study are available on request from the corresponding author.
